# Two Membrane-Associated Tyrosine Phosphatase Homologs Potentiate C. elegans AKT-1/PKB Signaling

**DOI:** 10.1371/journal.pgen.0020099

**Published:** 2006-07-07

**Authors:** Patrick J Hu, Jinling Xu, Gary Ruvkun

**Affiliations:** 1 Department of Molecular Biology, Massachusetts General Hospital, Department of Genetics, Harvard Medical School, Boston, Massachusetts, United States of America; 2 Division of Hematology/Oncology, Massachusetts General Hospital, Department of Genetics, Harvard Medical School, Boston, Massachusetts, United States of America; Huntsman Cancer Institute, United States of America

## Abstract

Akt/protein kinase B (PKB) functions in conserved signaling cascades that regulate growth and metabolism. In humans, Akt/PKB is dysregulated in diabetes and cancer; in *Caenorhabditis elegans,* Akt/PKB functions in an insulin-like signaling pathway to regulate larval development. To identify molecules that modulate C. elegans Akt/PKB signaling, we performed a genetic screen for enhancers of the *akt-1* mutant phenotype *(eak)*. We report the analysis of three *eak* genes. *eak-6* and *eak-5/sdf-9* encode protein tyrosine phosphatase homologs; *eak-4* encodes a novel protein with an *N*-myristoylation signal. All three genes are expressed primarily in the two endocrine XXX cells, and their predicted gene products localize to the plasma membrane. Genetic evidence indicates that these proteins function in parallel to AKT-1 to inhibit the FoxO transcription factor DAF-16. These results define two membrane-associated protein tyrosine phosphatase homologs that may potentiate C. elegans Akt/PKB signaling by cell autonomous and cell nonautonomous mechanisms. Similar molecules may modulate Akt/PKB signaling in human endocrine tissues.

## Synopsis

Insulin and insulin-like growth factor (IGF) signaling regulates critical physiological processes in a wide variety of multicellular organisms. In humans, dysregulation of IGF signaling underlies the pathogenesis of cancer and diabetes. In the nematode *Caenorhabditis elegans,* the DAF-2 insulin-like pathway regulates development, metabolism, and longevity. All known components of DAF-2 insulin-like signaling are structurally and functionally conserved in mammals, suggesting that insights gained from studying this pathway in C. elegans may shed light on pathogenetic mechanisms underlying cancer and diabetes. In this study, the authors describe a genetic screen designed to identify novel components of DAF-2 insulin-like signaling in C. elegans. They have characterized three genes that may encode parts of a novel multimolecular membrane-associated complex that potentiates DAF-2 insulin-like signaling in two neuroendocrine cells, the XXX cells. Two of these genes encode proteins similar to mammalian protein tyrosine phosphatases. These results suggest that protein tyrosine phosphatase–like molecules may transduce IGF signals in mammalian endocrine cells and highlight the role of endocrine circuits in the pathogenesis of cancer and diabetes.

## Introduction

Conserved signaling cascades activated by insulin and insulin-like growth factors (IGFs) are critical to the normal development, growth, and physiology of many organisms. In mice, insulin signaling regulates embryonic growth and glucose uptake [1], and insulin-like growth factor-1 (IGF-1) signaling regulates normal prenatal and postnatal growth [2]. Insulin-like signaling regulates cell size in *Drosophila* [3–5] and development, metabolism, and longevity in Caenorhabditis elegans [6–9].

In humans, dysregulation of insulin and IGF-1 signaling plays a prominent role in disease pathogenesis. Patients with type 2 diabetes mellitus exhibit resistance to insulin [10]; similar insulin resistance is observed in mice harboring mutations in the insulin receptor and downstream components of insulin signaling [11–14]. Downstream components of IGF-1 signaling have been implicated in cancer pathogenesis based on the identity of homologous transforming retroviral oncoproteins [15,16] as well as the existence of gene amplifications [17–19] and somatic mutations [20–23] in primary tumors and tumor cell lines.

Binding of IGFs to their cognate transmembrane receptors activates a cascade that is conserved throughout metazoan phylogeny [24–26]. In C. elegans this pathway includes 38 insulin-like proteins [9,27], an insulin/IGF-1-receptor–like molecule (DAF-2 [7]), PI 3-kinase catalytic (AGE-1 [28]) and adaptor (AAP-1 [29]) subunits, a phosphoinositide-dependent kinase (PDK-1 [30]), two Akt/protein kinase B (PKB) homologs (AKT-1 and AKT-2 [31]), and a serum- and glucocorticoid-inducible kinase homolog (SGK-1 [32]) (see later). Although the biological role of most of the insulins has not been established, a mutation in the *daf-28* insulin gene causes decreased insulin signaling [8], implicating DAF-28 as a candidate ligand for DAF-2/InsR (insulin receptor homolog) [9]. Additionally, the C. elegans INS-6 insulin can bind to and activate the human InsR tyrosine kinase [33]. Analogous to insulin and IGF-1 signaling in mammals [34,35], activation of DAF-2/InsR leads to the phosphorylation, cytoplasmic retention, and inhibition of the FoxO transcription factor DAF-16 [36–40]. DAF-2/InsR signaling is likely downregulated by the PTEN (phosphatase and tensin homolog) tumor suppressor homolog DAF-18 [41–44].

*daf-2/InsR* mutants were first identified based on their increased tendency to enter an alternative larval developmental stage called the dauer stage (*daf* refers to a dauer formation phenotype) [45]. In replete growth conditions, C. elegans undergoes four larval molts prior to reaching reproductive adulthood [46]. Under conditions of high population density, high temperature, or starvation, early larvae bypass the normal second and third larval stages and instead develop into the alternative dauer larva. Dauers are morphologically distinct from normal L3 larvae, exhibiting radial and pharyngeal constriction, decreased pharyngeal pumping, and cuticular specializations called alae. In addition, they increase intestinal fat storage and exhibit extended longevity. Upon improvement of ambient conditions, dauers recover to the L4 larval stage and proceed to reproductive adulthood [47].

Genetic screens have defined three signaling pathways that normally function to inhibit dauer arrest under replete environmental conditions [45,48–50]. In addition to DAF-2/InsR inputs, dauer arrest is regulated by conserved DAF-7/transforming growth factor (TGF)-β–like [51] and DAF-11/guanylyl cyclase [52] signaling pathways. Insulin and TGF-β–like ligands are expressed in small subsets of head neurons, and this expression is regulated by various environmental inputs and by cyclic guanosime monophosphate signaling [9,27,51,53]. These ligands likely bind to cognate receptors that are expressed widely in target tissues throughout the animal [54]. The major targets of DAF-7/TGF-β signaling are the SMAD4 tumor suppressor homolog DAF-3 [55] and its binding partner DAF-5/SNO [56,57]. Mutations in *daf-16/FoxO* specifically suppress dauer formation of *daf-2/InsR* pathway mutants, indicating that DAF-16/FoxO is the major target of DAF-2/InsR signaling in C. elegans [49,50]. Genetic analysis indicates that these pathways function in parallel to promote normal larval development [48,49].

Although many components of DAF-2/InsR signaling have been characterized, at least three lines of evidence indicate that undiscovered pathway components that act downstream of DAF-2/InsR but parallel to AGE-1/PI3K may exist. Gain-of-function mutations in *pdk-1* and *akt-1* suppress the dauer-constitutive phenotype of *age-1/PI3K* null mutants more efficiently than they do that of *daf-2/InsR* mutants [30,31]. The weak *daf-18/PTEN(e1375)* allele suppresses dauer arrest in *age-1/PI3K* null mutants [42,43,49] but does not suppress the dauer-constitutive phenotype of *daf-2(e1370)* mutants [42,43,50]. Last, a functional DAF-16::GFP fusion protein harboring point mutations in all four putative Akt/PKB phosphorylation sites localizes to the nucleus but does not increase life span or induce dauer formation in a *daf-2* wild-type background [38].

The tumor suppressor function of the 3-phosphoinositide phosphatase PTEN [20,21], the frequent somatic mutation of phosphatidylinositol (PI) 3-kinase in human cancers [23], and the discovery of a germline Akt2 loss-of-function mutation in a family with autosomal dominant insulin-resistant diabetes [58] underscore the central role of this pathway in human disease. In view of the striking structural and functional conservation of insulin-like signaling throughout metazoan phylogeny [59], it is likely that the identification of novel DAF-2/InsR signaling components will illuminate not only mechanisms of developmental regulation in C. elegans but also the pathogenesis of common human diseases such as cancer and diabetes.

To identify genes encoding such molecules, we performed a genetic screen for mutants that enhance the dauer-constitutive phenotype of an *akt-1* null mutant (Eak screen). Seven genetic loci enhance *akt-1* when mutated; we report the molecular identity of three *eak* loci here. These genes may encode elements of a membrane-associated complex that functions in two endocrine cells to potentiate insulin-like signaling. A similar activity may modulate insulin and IGF-1 signals in human endocrine tissues.

## Results

### A Sensitized Genetic Screen for Akt/PKB Signaling Components

**Figure 1:**
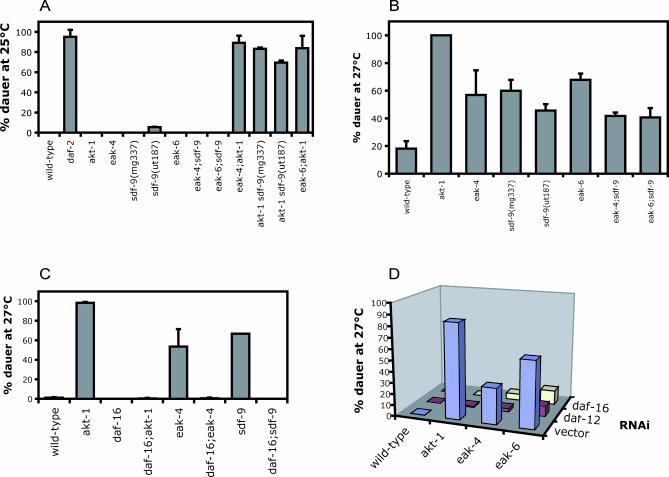
Dauer Formation Phenotypes of *eak* Mutants *eak* single mutants, *eak-x;eak-y* double mutants, or *eak;akt-1* double mutants were assayed for dauer arrest at (A) 25 °C and (B) 27 °C. (C) *eak* 27 °C dauer arrest phenotypes are suppressed by a mutation in *daf-16/FoxO*. (D) *akt-1, eak-4,* and *eak-6* 27 °C dauer arrest phenotypes are suppressed by RNAi of *daf-16/FoxO* and *daf-12/NHR*. All error bars indicate standard deviation. All experiments were performed three times. Refer to Table S1 for numbers of animals scored. *eak-5* is allelic to the synthetic dauer formation gene *sdf-9* [62] and is referred to as *sdf-9* throughout the paper. Mutant alleles used were *daf-2(e1370), akt-1(mg306), eak-4(mg348), sdf-9(mg337)* and *sdf-9(ut187), eak-6(mg329),* and *daf-16(mgDf47)*. *sdf-9(ut187)* was used to construct the *eak-4;sdf-9, eak-6;sdf-9,* and *daf-16;sdf-9* double mutants. Multiple alleles of *eak-4* and *sdf-9* yielded phenotypes similar to those shown. See Table S1 for numbers of animals assayed.

We hypothesized that a genetic screen performed in a weak dauer-constitutive *daf-2/InsR* pathway mutant background might allow the identification of DAF-2/InsR signaling components which, when mutated alone, might not have phenotypes. *akt-1(mg306)* was isolated in a genetic screen for mutations that affect the production of or response to elevated 3-phosphoinositide levels and contains a C→T transition that generates a nonsense mutation in the pleckstrin homology domain (see Materials and Methods). In contrast to *daf-2/InsR, age-1/PI3K,* and *pdk-1* loss-of-function *(lf)* mutants, which form dauers at 25 °C or lower [28,30,60], *akt-1(lf)* alleles have a Hid (high-temperature-induced dauer) phenotype [61], developing reproductively at 25 °C but forming dauers at 27 °C (unpublished data and Figure 1). Consistent with a role for AKT-1 in DAF-2/InsR signal transduction [31,43], the 27 °C dauer-constitutive phenotype of *akt-1* mutants is suppressed by a *daf-16/FoxO* (*lf*) mutation [61].

We mutagenized *akt-1(mg306)* animals and screened for dauer arrest at 25 °C, a temperature at which *akt-1(mg306)* mutants do not form dauers (Figure 1A). Among 30 independent mutants isolated from approximately 21,000 haploid genomes screened, 26 were suppressible by *daf-16/FoxO* feeding RNAi (unpublished data), suggesting that their dauer-constitutive phenotypes were either dependent upon the presence of the *akt-1(mg306)* mutation or the result of mutations in the *daf-2/InsR* pathway [49,50], or both. Twenty-one of these 26 mutants were true Eak mutants, exhibiting dauer arrest at 25 °C only in a homozygous *akt-1(mg306)* background (unpublished data and Figure 1A). Mapping and complementation analysis indicate that these 21 mutants define seven Eak genes.

All *eak;akt-1(mg306)* double mutants formed a high percentage of dauers at 25 °C (Figure 1A), exhibiting a dauer-constitutive phenotype as strong as the canonical *daf-2/InsR* mutant *daf-2(e1370)*. However, in contrast to *daf-2(e1370)* dauers, which have constricted pharynxes and dauer-specific cuticular alae, all *eak* and *eak;akt-1(mg306)* dauers were partial dauers, exhibiting characteristic alae but failing to undergo pharyngeal remodeling (Figure S1). *eak* mutants also enhanced dauer arrest phenotypes of weak alleles of the *daf-2/InsR* pathway components *age-1/PI3K* and *pdk-1;* whereas *age-1(hx546)* and *pdk-1(sa709)* did not exhibit dauer arrest at 25 °C, *age-1;eak* and *eak;pdk-1* double mutants had strong dauer arrest phenotypes at 25 °C (Figure S2A). Notably, *eak* mutants did not enhance dauer arrest phenotypes of *akt-2(ok393),* a deletion allele of *akt-2* (Figure S2B). Therefore, *eak* gene products may function in the same pathway as AKT-2.

In an *akt-1*(+) background, *eak* mutants had minimal dauer-constitutive phenotypes at 25 °C (Figure 1A) and relatively weak dauer-constitutive phenotypes in comparison to *akt-1(mg306)* animals at 27 °C (Figure 1B). The ability of *eak* mutants to enhance dauer arrest phenotypes of *age-1/PI3K, akt-1,* and *pdk-1* loss-of-function mutants is consistent with their functioning in a parallel pathway, and their low penetrance dauer arrest phenotype at 25 °C would explain why these mutants were not isolated in previous screens for dauer arrest mutants performed at this temperature.

### The *eak* Genes Act in the C. elegans Insulin-like Pathway

Loss-of-function mutations in *daf-16/FoxO* specifically suppress the dauer arrest phenotype of *daf-2/InsR* pathway mutants [49,50]. To determine whether *eak* gene products function in the *daf-2/InsR* pathway, we performed epistasis analysis on *daf-16;eak* double mutants and *daf-16;eak;akt-1* triple mutants. For epistasis analysis of *eak-6(mg329)*, we used feeding RNAi of *daf-16/FoxO;* close linkage of *eak-6* and *daf-16* on Chromosome I prevented construction of an *eak-6 daf-16* double mutant. A *daf-16/FoxO* null mutation, *mgDf47* [36], fully suppressed dauer-constitutive phenotypes of all *eak* single mutants tested (Figure 1C) and both *eak;akt-1* double mutants tested (Figure S3A), whereas a *daf-3/SMAD* null mutation did not [62]. RNAi of *daf-16/FoxO* also suppressed *akt-1(mg306), eak-4(mg348),* and *eak-6(mg329)* dauer-constitutive phenotypes, as did RNAi of the nuclear hormone receptor gene *daf-12* (Figure 1D; loss-of-function *daf-12* mutations suppress dauer arrest caused by most known dauer-constitutive mutants [48,50,61,63–65]). These findings suggest that *eak* genes function in the *daf-2/InsR* pathway.

To help determine whether *eak* gene products function in parallel to AKT-1, we constructed *eak;daf-18* and *eak;akt-1* gain-of-function double mutants and performed epistasis analysis. The weak *daf-18/PTEN* allele *e1375* suppresses dauer arrest in *age-1/PI3K* null mutants but does not suppress dauer arrest in *daf-2(e1370)* [42,43,49,50], and the *akt-1* gain-of-function allele *mg144* exhibits stronger suppression of *age-1/PI3K* null phenotypes than *daf-2/InsR* loss-of-function phenotypes [31], suggesting that DAF-18/PTEN and AKT-1 function in an AGE-1/PI3K–specific branch of DAF-2/InsR outputs. An inability of *daf-18(e1375)* or *akt-1(mg144)* to suppress *eak* dauer arrest would indicate that *eak* gene products function in parallel to and independently of AGE-1/PI3K. Both *daf-18(e1375)* and *akt-1(mg144)* strongly suppressed dauer arrest in *eak-4, eak-5/sdf-9,* and *eak-6* mutants (Figure S3B and S3C), suggesting that *eak* gene products function either upstream of or in parallel to AGE-1/PI3K and AKT-1.

To address the issue of whether all three *eak* gene products function in the same pathway, we constructed *eak-x;eak-y* double mutants and tested them for dauer arrest at 25 °C and 27 °C. Lack of enhanced dauer arrest in double mutants would suggest that *eak* gene products function together in the same complex or pathway. *eak-4;sdf-9* and *eak-6;sdf-9* double mutants did not arrest as dauers at 25 °C (Figure 1A) and did not exhibit enhanced dauer arrest at 27 °C (Figure 1B), indicating that the EAK proteins likely function together in the same complex or pathway.

Most dauer-constitutive mutants in the *daf-2/InsR* pathway also have extended organismal longevity [6,29,30,66]. Therefore, we performed longevity assays on all *eak* mutants. Consistent with a recent report [32], the *akt-1(mg306)* mutation extended median life span by less than 10%, which is a smaller extension of life span than has been seen with other loss-of-function dauer-constitutive mutants in the *daf-2/InsR* pathway. Interestingly, no *eak* mutants tested extended median or maximum life span significantly (Figure S4). Furthermore, *eak* alleles did not enhance life span extension of *akt-1(mg306),* although they all strongly enhanced the dauer formation phenotype of *akt-1(mg306)* (Figure 1A). Thus, *eak* signaling does not subserve longevity control.

### Two *eak* Genes Are Related to Protein Tyrosine Phosphatases

Nine mutants define the *eak-5* gene. Single nucleotide polymorphism (SNP) mapping [67] localized *eak-5* to an approximately 480-kb genomic interval between cosmid F48F5 and the right telomere of Chromosome V. *sdf-9,* identified in a screen for enhancers of the dauer-constitutive phenotype of *unc-31(e169)* [62], lies within this interval. Sequencing of *sdf-9* exons and splice junctions in all nine *eak-5* alleles identified eight distinct point mutations (Table 1), and an *akt-1 sdf-9* double mutant constructed using the *sdf-9(ut187)* allele identified in the *unc-31(e169)* enhancer screen [62] had a strong dauer-constitutive phenotype at 25 °C (Figure 1A), indicating that *eak-5* is allelic to *sdf-9*.

**Table 1:**
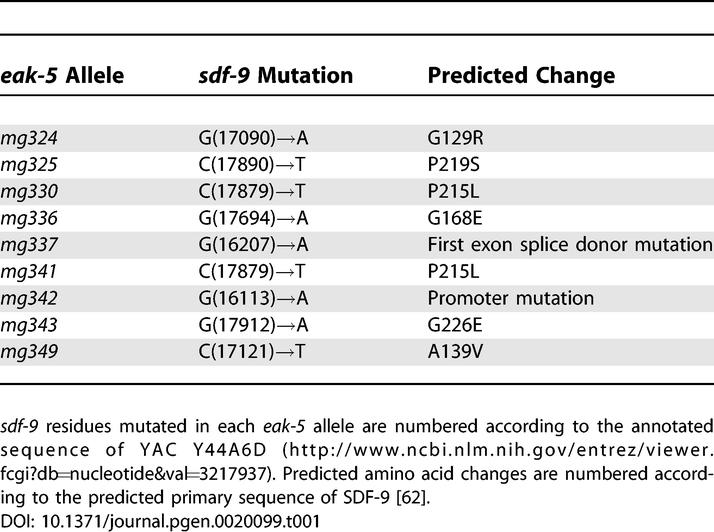
*eak-5* Is Allelic to the Synthetic Dauer Formation Gene *sdf-9*

**Figure 2:**
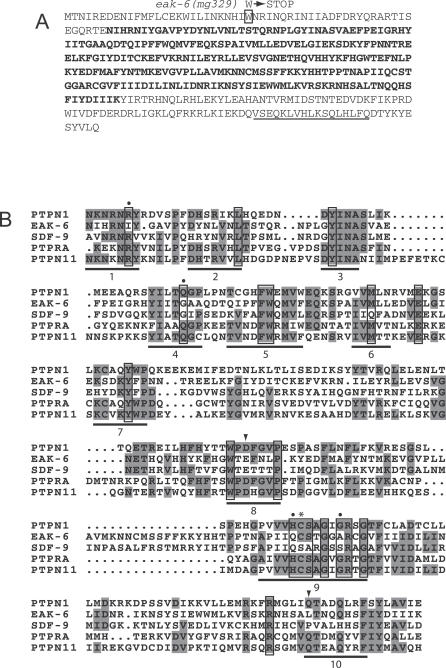
Predicted Primary Amino Acid Sequence of EAK-6 and Similarity with PTPs (A) EAK-6 amino acid sequence. EAK-6 sequence was derived from full-length cDNA amplified by RT-PCR from wild-type C. elegans total RNA. The PTP domain is denoted in boldface. The residue mutated in *eak-6(mg329)* is boxed and the predicted change indicated above the mutated residue. Amino acids encoded by an alternatively spliced exon present in EAK-6L but not in EAK-6S (see Materials and Methods) are underlined. (B) EAK-6 homology with PTPs. The PTP domain of EAK-6 is aligned with that of SDF-9 and 3 human PTPs. Sequences used in the alignment are based on domains defined by Pfam [101] and correspond to amino acids 40 to 276 of PTPN1 (PTP1B), 57 to 308 of EAK-6, 31 to 283 of SDF-9, 265 to 500 of PTPRA (receptor-type PTPα), and 273 to 520 of PTPN11 (SHP-2). Alignment was performed using ClustalX 1.8 and MacBoxshade 2.15. Conserved and identical residues are shaded. Ten motifs conserved among 37 vertebrate PTPs [68] are underlined, and 19 residues that are invariant among 113 vertebrate PTP domains are boxed. The four invariant residues that are not identical in EAK-6 (R45, Q85, H214, and G220, numbered according to the PTPN1 primary sequence) are denoted with dots. The catalytic cysteine residue [69] is denoted by an asterisk. Two conserved residues that are not conserved in EAK-6, D181 and Q262, are denoted by arrowheads.

The *eak-6* gene is defined by one allele, *mg329*. SNP mapping localized *eak-6(mg329)* to an approximately 210-kb genomic region on Chromosome I between cosmids F52F12 and B0379. BLASTP analysis of SDF-9 against the C. elegans Wormpep database (http://www.sanger.ac.uk/Projects/C_elegans/WORMBASE/current/wormpep.shtml) identified a predicted homolog encoded by open reading frame F10G8.4. This open reading frame lies within the *eak-6* genomic interval defined by our SNP mapping. Sequencing of predicted exons and splice junctions of F10G8.4 in *eak-6(mg329)* identified a G→A transition resulting in an opal nonsense mutation near the predicted amino-terminus of the protein (Figure 2A). Two of nine transgenic lines containing a genomic PCR fragment including the putative promoter, open reading frame, and 3' untranslated regions (UTR) of F10G8.4 exhibited rescue of the dauer arrest phenotype of an *eak-6(mg329);akt-1(mg306)* double mutant (unpublished data), supporting the argument that F10G8.4 is *eak-6*.

EAK-6 and SDF-9 both have amino acid similarity to PTPs (Figure 2B and [62]). Conservation is strongest in the ten motifs that are conserved among 37 human PTPs [68]. Whereas SDF-9 does not retain the canonical catalytic cysteine residue found in all known PTPs and is therefore predicted to be catalytically inactive [62,69], the catalytic cysteine is conserved in EAK-6. Despite this difference, *sdf-9* and *eak-6* mutants have a similar phenotype (Figure 1A and 1B). Among 19 amino acid residues that are invariant among 113 vertebrate PTPs [68], 15 are conserved in EAK-6 and 13 are conserved in SDF-9 (Figure 2B). PTP activity assays on both epitope-tagged EAK-6 expressed in and immunoprecipitated from cultured human cells and a GST-EAK-6 fusion protein expressed in Escherichia coli revealed no hydrolytic activity on the substrate *p*-*nitro-*phenylphosphate (PNPP), a phosphotyrosine analog (unpublished data). Thus, EAK-6 and SDF-9 may be inactive phosphatase homologs that bind to tyrosine phosphoproteins. However, we cannot rule out the possibility that EAK-6 has PTP activity that is not detectable in the PNPP assay.

### *eak-4* Encodes a Novel Protein with an *N*-Myristoylation Signal

**Figure 3:**
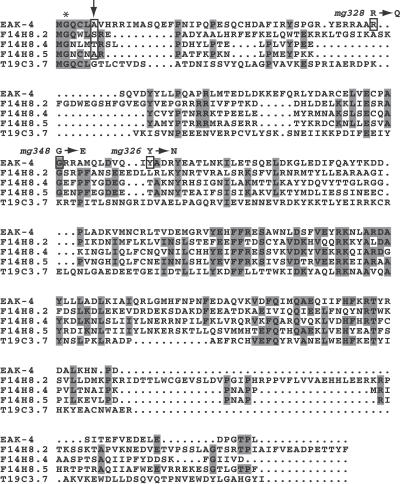
EAK-4 Amino Acid Sequence and Alignment with Four C. elegans Homologs Sequences represent the entire predicted amino acid sequences of all five genes. EAK-4 sequence was derived from full-length cDNA amplified by RT-PCR from wild-type C. elegans total RNA. Alignment was constructed using ClustalX 1.8 and MacBoxshade 2.15. Shaded residues indicate amino acid identity and conservation. Mutated residues in three alleles of *eak-4* are boxed, and the predicted amino acid change is indicated above the box. The conserved G at residue 2 and S/T/A (boxed) at residue 6 in the *N*-myristoylation consensus sequence [70] are denoted by an asterisk and a vertical arrow, respectively.

Three alleles, *mg326, mg328,* and *mg348,* define the *eak-4* locus. SNP mapping narrowed the *eak-4* interval to an approximately 560-kb genomic region on Chromosome IV between cosmids B0001 and T23B5. Cosmid rescue assays identified F53B2 as the rescuing cosmid, and rescue experiments with genomic PCR fragments corresponding to predicted genes on F53B2 identified open reading frame F53B2.3 as a candidate gene for *eak-4*. Sequencing of predicted exons and splice junctions in F53B2.3 identified independent point mutations in all three *eak-4* alleles (Figure 3). RT-PCR of wild-type C. elegans total RNA confirmed the WormBase (WormBase Web site, http://www.wormbase.org, release WS120, March 1, 2004) predicted mRNA structure. A PCR fragment containing the predicted F53B2.3 promoter, open reading frame, and 3' UTR rescued the *eak-4* mutant phenotype in one of one transgenic line, further supporting the conclusion that F53B2.3 is *eak-4*.

EAK-4 has amino acid similarity to four other C. elegans proteins, denoted by cosmid gene names F14H8.2, F14H8.4, F14H8.5, and T19C3.7 (Figure 3). The function of these proteins is not known. A glycine residue that is conserved among EAK-4, F14H8.2, F14H8.4, and F14H8.5 is mutated in *mg348,* which is the strongest *eak-4* allele based upon the 20 °C dauer-constitutive phenotype of *eak-4;akt-1* double mutants (unpublished data). A heteroallelic strain with *eak-4(mg348) in trans* to a deficiency did not exhibit enhanced dauer arrest (Figure S5), suggesting that *eak-4(mg348)* is a null allele. Notably, EAK-4, F14H8.2, F14H8.4, and F14H8.5 all have canonical *N*-myristoylation sequences [70], suggesting that they may associate with membranes.

### *eak* Promoters Drive Transcription Specifically in the XXXL/R Cells

A functional SDF-9::GFP fusion protein is expressed specifically in the XXXL/R cells [62]. Promoter fusions of *eak-4* and *eak-6* to GFP [71] were specifically expressed in two cells in the head that we identified as the XXXL/R cells based on their variable positions and neurite-like morphology [62]. Most *eak-6p::GFP*-containing animals also exhibited GFP expression in a third cell identified as the pharyngeal M1 motor neuron based on the position of its cell body and axonal process [72].

**Figure 4:**
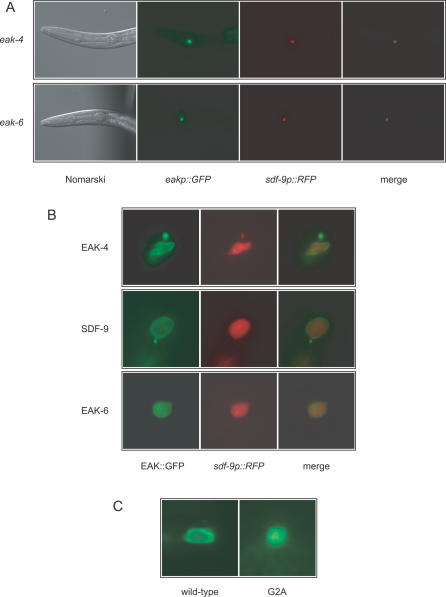
EAK-4, SDF-9, and EAK-6 Localize to the Plasma Membrane of the XXX Cells (A) *eak-4, sdf-9,* and *eak-6* promoters drive expression in the same cells. Wild-type animals harboring an extrachromosomal array with *eakp::GFP* and *sdf-9p::RFP* constructs were analyzed by fluorescence microscopy. Representative photographs are shown. Animals are oriented anterior left and dorsal up. (B) EAK-4::GFP, SDF-9::GFP, and EAK-6::GFP fusion proteins localize to the plasma membrane of XXX. Animals harboring EAK::GFP translational fusion constructs and an integrated *sdf-9p::RFP* array were analyzed using fluorescence microscopy. Representative photographs of a XXX cell are shown. (C) Mutation of the invariant glycine in the *N*-myristoylation motif of EAK-4 abrogates plasma membrane localization. Animals harboring either a wild-type EAK-4::GFP construct or an EAK-4::GFP construct with the glycine at position 2 mutated to alanine (G2A) were analyzed using fluorescence microscopy.

To confirm this result, we constructed an *sdf-9* promoter fusion to red fluorescent protein [73], coinjected either *eak-4p::GFP* or *eak-6p::GFP* with *sdf-9p::RFP,* and assayed for colocalization of GFP and RFP in transgenic animals. The *sdf-9p::RFP* fusion was expressed exclusively in two head cells with position and morphology consistent with their identification as the XXX cells [62]. In both strains containing GFP and RFP reporter constructs, GFP and RFP colocalized (Figure 4A), indicating that *eak-4, sdf-9,* and *eak-6* are all expressed in XXXL/R.

### EAK-4, SDF-9, and EAK-6::GFP Fusion Proteins Localize to the Plasma Membrane

In order to visualize the subcellular localization of EAK proteins, we made full-length translational GFP fusion constructs and expressed them in wild-type animals. Both EAK-4::GFP and EAK-6::GFP fusion proteins were expressed specifically in XXX, as was an SDF-9::GFP fusion protein [62]. They were also variably expressed in the intestine (unpublished data), a common site of artifactual GFP expression [74,75]. Coexpression of translational GFP fusions with an *sdf-9p::RFP* promoter fusion indicated that all three GFP fusions localize to the plasma membrane of the XXX cells (Figure 4B). Membrane localization was also apparent in intestinal cells (unpublished data).

We determined the role of the *N*-myristoylation consensus motif in EAK-4 plasma membrane localization by constructing an EAK-4::GFP mutant in which the invariant glycine residue at position 2 is mutated to alanine (G2A). In contrast to wild-type EAK-4::GFP, which was localized to the plasma membrane (Figure 4B and 4C), the EAK-4 G2A mutant GFP fusion protein exhibited diffuse cytoplasmic localization (Figure 4C), indicating that an intact *N*-myristoylation motif is required for EAK-4 plasma membrane localization.

To gain insight into the influence of DAF-2/InsR signaling on EAK plasma membrane localization, we examined SDF-9::GFP subcellular localization in *daf-2(e1370)* mutant animals grown at 25 °C. At this temperature, *daf-2(e1370)* animals undergo dauer arrest (Figure 1A). SDF-9::GFP exhibited plasma membrane localization in both wild-type and *daf-2(e1370)* animals (Figure S6), indicating that its localization does not require normal levels of DAF-2/InsR signaling.

### Expression of AKT-1::GFP in XXXL/R Rescues Dauer Arrest in an *eak-4;akt-1* Double Mutant

**Figure 5:**
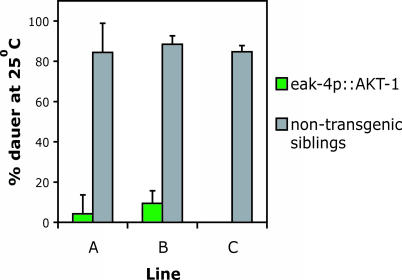
Expression of AKT-1 in XXX is Sufficient to Rescue the 25 °C Dauer Phenotype of an *eak-4;akt-1* Double Mutant *eak-4(mg326);akt-1(mg306)* double mutant animals carry a transgene containing the *akt-1* genomic region and 3' untranslated region under the control of the *eak-4* promoter. Three independent lines rescue *eak-4(mg326);akt-1(mg306)* dauer arrest at 25 °C. Error bars indicate standard deviation. This experiment was performed twice. Refer to Table S1 for numbers of animals scored.

In stark contrast to the specific expression of EAK::GFP proteins in the XXX cells (Figure 4A and 4B), a functional AKT-1::GFP fusion protein is expressed widely in postembryonic animals [31]. To determine the importance of AKT-1 function specifically in XXXL/R, we expressed the *akt-1* open reading frame and 3' UTR under the control of the *eak-4* promoter and asked whether this transgene could rescue dauer arrest in an *eak-4(mg326);akt-1(mg306)* double mutant. In three of three transgenic lines assayed, animals harboring the *eak-4p*::AKT-1 transgene bypassed dauer arrest and grew reproductively, whereas nontransgenic siblings formed dauers (Figure 5). Control transgenic animals expressing an *eak-4p*::GFP transcriptional fusion construct did not bypass dauer (unpublished data). These results show that AKT-1 expression in the XXX cells is sufficient to rescue the *akt-1(mg306)* dauer arrest phenotype and indicate that the XXX cells are a major site of AKT-1 function in C. elegans.

### A DAF-16A::GFP Fusion Protein Is Not Expressed in XXXL/R

**Figure 6:**
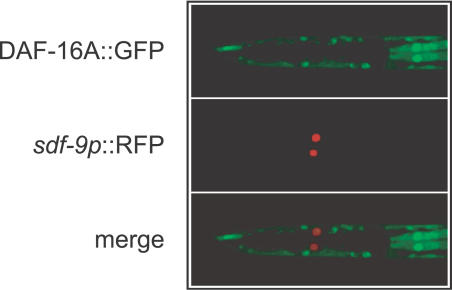
A Functional DAF-16A::GFP Fusion Protein Is Not Expressed in XXX Wild-type animals harboring integrated DAF-16A::GFP and *sdf-9p*::RFP arrays were analyzed by confocal microscopy. Representative photographs of a single animal are shown. The animal is oriented anterior left and dorsal up. Merging of GFP and RFP images reveals no colocalization of fluorescent proteins.

In order to assess whether *eak* gene products regulate DAF-16/FoxO cell autonomously, we first determined whether a functional DAF-16A::GFP fusion protein under the control of the *daf-16a* promoter is expressed in XXXL/R. We constructed a strain harboring integrated DAF-16A::GFP and *sdf-9p*::RFP transgenes and assayed for colocalization of GFP and RFP. Surprisingly, although DAF-16A::GFP is widely expressed [40], it did not colocalize with RFP (Figure 6), indicating that it is not expressed at high levels in XXXL/R. Therefore, *eak* gene products may regulate DAF-16/FoxO nonautonomously.

## Discussion

We have used an *akt-1* enhancer screen to identify three genes encoding proteins that potentiate AKT-1 signaling. It is noteworthy that this screen has yielded neither weak alleles of known dauer-constitutive genes encoding components of DAF-2/InsR, DAF-7/TGF-β, or DAF-11/GC pathways nor strong alleles of the many mutants with weak dauer-constitutive phenotypes [61,63,76]. The fact that we have isolated multiple alleles of five of the seven genes identified in our screen (Figure 3, Table 1, and unpublished data) without identifying alleles of most mutants with weak dauer phenotypes argues that the target for *akt-1* enhancement is very specific and that most mutants with weak dauer phenotypes do not strongly enhance *akt-1(mg306)*. Therefore, *eak-4, sdf-9,* and *eak-6* likely do not enhance *akt-1(mg306)* simply by virtue of their weak dauer-constitutive phenotype; rather, these genes probably encode components of a complex or pathway that cooperates specifically with AKT-1.

*sdf-9* was also identified in a genetic enhancer screen using the weak dauer arrest mutant *unc-31(e169)* as a genetic background [62]. *unc-31* encodes a homolog of CAPS, a protein required for calcium-induced dense core vesicle exocytosis [77]. Since insulin is stored in and secreted from dense core vesicles in pancreatic islet beta cells [78], it is likely that UNC-31/CAPS and AKT-1 function in the same insulin signaling pathway. This is underscored by the suppression of the weak dauer arrest phenotypes of *akt-1* and *unc-31* mutants by *daf-16/FoxO* mutations (Figure 1C and 1D [61]) and may explain why *sdf-9* mutants were isolated in both screens. *akt-1 sdf-9* and *unc-31;sdf-9* double mutants have similar dauer arrest phenotypes at 25 °C (Figure 1A and [62]).

Surprisingly, we have not identified any *akt-2* mutants in this screen. We expected *akt-2* mutants to emerge from this screen, given that *akt-1;akt-2* double mutants [79] as well as animals subjected to simultaneous RNAi of *akt-1* and *akt-2* [31,32] undergo dauer arrest. Furthermore, *eak* mutants do not enhance dauer arrest of *akt-2* mutants (Figure S2B), indicating that they may act in the same pathway. However, none of the 21 alleles isolated in the Eak screen is X-linked (the *akt-2* gene lies on the X chromosome). In the course of constructing an *akt-1(mg306);akt-2(ok393)* double mutant, we noted that *akt-1;akt-2* animals formed nonconditional dauers in the F_3_ generation but were maternally rescued for dauer arrest in the F_2_ generation (unpublished data), explaining why *akt-2* alleles were not isolated in this F_2_ screen. It is possible that other components of DAF-2/InsR signaling would emerge from an F_3_ *akt-1* enhancer screen.

In contrast to previous results demonstrating a strong dauer arrest phenotype for multiple *sdf-9* alleles [62], we have only observed weak dauer phenotypes for all *eak-4, sdf-9,* and *eak-6* alleles tested (Figure 1 and unpublished data). The alleles tested included the *ut163* and *ut187* alleles of *sdf-9* previously described (Figure 1A and 1B; [62]). As dauer assays at 27 °C are exquisitely sensitive to small changes in environmental conditions [63], we attribute these differences to small discrepancies between assay conditions in different laboratories. This may also explain the disparate effects of XXX laser ablation on dauer formation reported in the literature [62,65,80].

### The XXX Cells as a Site of DAF-2/InsR Function

The XXX cells are annotated as hypodermal cells in the head [81] that abut the pseudocoelom and have neuronal characteristics [62,80,82]. Laser ablation of the XXX cells causes partial dauer arrest [62], indicating that the XXX cells normally function to inhibit dauer arrest. The specific expression of EAK-4, SDF-9, and EAK-6 in the XXX cells (Figure 4), taken together with our finding that AKT-1 expression in XXX suffices to rescue the dauer arrest phenotype of an *eak-4;akt-1* double mutant (Figure 5), indicates that the XXX cells may be a major site of DAF-2/InsR function in C. elegans. However, the output of the XXX cell in the regulation of dauer arrest also depends on insulin signaling in other cells, since the dauer arrest phenotype caused by ablation of the XXX cells is suppressed by mutations in *daf-16/FoxO* but not by mutations in *daf-3/SMAD4* [62], This is also consistent with our observation that DAF-16::GFP expressed from the *daf-16a* promoter is not expressed in XXX (Figure 6). It is not known whether DAF-2/InsR is expressed in XXX.

As reported previously for *sdf-9* [62], we have observed that *eak-4, sdf-9,* and *eak-6* single mutants form partial dauers that have cuticular alae but have not undergone pharyngeal remodeling (Figure S1). Indeed, some *daf-2/InsR* mutant alleles also cause partial dauer arrest [60]. It is possible that signals from the XXX cells control dauer arrest and extrapharyngeal remodeling, whereas DAF-2/InsR signaling outputs from other cells, for example, the pharyngeal tissues themselves, may affect pharyngeal remodeling. Phenotypes of rare *daf-2* mosaics that lack *daf-2* activity in both XXX cells and various portions of the ABa cell lineage [83] suggest that descendants of the ABalpa cell may regulate pharyngeal remodeling during dauer formation.

### EAK-4, SDF-9, and EAK-6 May Be Novel Components of DAF-2/InsR Signaling

Enhancement of *akt-1* phenotypes could occur either through further attenuation of DAF-2/InsR signaling or by derangement of DAF-7/TGF-β or DAF-11/GC signaling [36]. As is the case for all known components of the DAF-2/InsR pathway [30–32, 48, 49], *eak-4, sdf-9,* and *eak-6* phenotypes are fully suppressed by mutations in *daf-16/FoxO* (Figure 1C and 1D [62]), the major target of DAF-2/InsR signaling [37,49]. This supports the contention that EAK-4, SDF-9, and EAK-6 are components of DAF-2/InsR signaling. Indeed, the strong, nonconditional dauer phenotype of some *eak;akt-1* double mutants (unpublished data) is reminiscent of the phenotype of strong loss-of-function alleles of *daf-2/InsR* [60].

Results of epistasis experiments with the weak *daf-18/PTEN* allele *e1375* [42,43] and the *akt-1* gain-of-function allele *mg144* [31] (Figure S3B and S3C) implicate EAK-4, SDF-9, and EAK-6 function upstream of or in parallel to AGE-1/PI3K and AKT-1. Since one of the molecular consequences of either DAF-18/PTEN loss-of-function or AKT-1 gain-of-function is phosphorylation and inhibition of DAF-16/FoxO [38–40], it is not surprising that *daf-18(e1375), akt-1(mg144),* and *daf-16(mgDf47)* are all epistatic to *eak* mutants.

### EAK Proteins May Inhibit DAF-16/FoxO Nonautonomously

*eak-4, sdf-9,* and *eak-6* mutants strongly enhance the *akt-1* null phenotype (Figure 1A), suggesting that EAK proteins function in parallel to AKT-1. In addition, *eak-4;sdf-9* and *eak-6;sdf-9* double mutants do not exhibit more severe phenotypes compared to the respective single mutants (Figure 1A and 1B), indicating that EAK-4, SDF-9, and EAK-6 function in the same pathway or complex. Parallel signaling of AKT-1 and EAK proteins could occur either at the organismal level, whereby AKT-1 signaling in non-XXX cells would converge with EAK signals in XXX to inhibit DAF-16/FoxO cell nonautonomously, or at the intracellular level, whereby AKT-1 signaling would converge with EAK signals in XXX to inhibit DAF-16/FoxO cell autonomously. The ability of AKT-1 expressed specifically in XXX to rescue an *eak-4;akt-1* double mutant (Figure 5) supports the notion that AKT-1 functions at least in part by signaling in parallel to EAK-4, SDF-9, and EAK-6 in XXX. However, the observation that dauer arrest caused by XXX laser ablation is suppressed by a *daf-16* loss-of-function mutant [62] and the lack of DAF-16A::GFP expression in XXX (Figure 6) suggest that EAK proteins regulate DAF-16A nonautonomously. It is possible that AKT-1 phosphorylates critical substrates in XXX distinct from DAF-16/FoxO. Alternatively, since there are at least three DAF-16/FoxO isoforms that may be transcribed from distinct promoters (WormBase Web site, http://www.wormbase.org, release WS150, November 30, 2005), a DAF-16/FoxO isoform distinct from DAF-16A may be a target of AKT-1 and EAK signals in XXX.

### Mechanisms of SDF-9 and EAK-6 Membrane Localization

In contrast to EAK-4, which likely localizes to the plasma membrane through *N*-myristoylation (Figure 4C), the mechanisms underlying the membrane localization of SDF-9 and EAK-6 are not clear. Neither SDF-9 nor EAK-6 possesses an *N*-myristoylation motif or a predicted transmembrane domain. SDF-9 and EAK-6 could associate with the plasma membrane by binding to membrane-associated proteins such as EAK-4. Alternatively, given that SDF-9 (and possibly EAK-6) has an inactive PTP domain that might bind to phosphotyrosine residues, they could bind to membrane-associated tyrosine phosphoproteins such as DAF-2/InsR. The ability of the DAF-18 ortholog PTEN to hydrolyze both phosphotyrosine [84] and phosphoinositides [85] suggests a third possible mechanism of SDF-9 and EAK-6 membrane localization via direct binding to phospholipids.

We addressed each model experimentally. First, coprecipitation assays on *N*-terminal epitope-tagged EAK-4, SDF-9, and EAK-6 expressed in all pairwise combinations in cultured 293T cells failed to reveal direct physical interactions among the three proteins despite high levels of protein expression (unpublished data). Furthermore, epitope-tagged SDF-9 and EAK-6 did not coprecipitate tyrosine phosphoproteins after exposure of transfected cultured 293T cells to IGF-1 (unpublished data). Finally, radiolabeled SDF-9 and EAK-6 synthesized in vitro did not bind to phosphoinositides immobilized on nitrocellulose (Seth Field and Lewis Cantley, personal communication). The observation that SDF-9::GFP exhibits plasma membrane localization in the absence of intact DAF-2/InsR signaling (Figure S6) is consistent with the lack of SDF-9 binding to tyrosine phosphoproteins in 293T cells and suggests that SDF-9 membrane association is independent of tyrosine phosphorylation. We cannot rule out the possibility that biologically relevant EAK protein-protein or protein-lipid interactions require additional components that exist in vivo but were not present in the assays described. Notably, one (or more) of the four *eak* genes that remain to be cloned *(eak-1, eak-2, eak-3,* and *eak-7)* may encode such a molecule.

### SDF-9 and EAK-6 Are Similar to Tyrosine Phosphatases

The similarity of SDF-9 and EAK-6 amino acid sequences to PTPs (Figure 2B and [62]) suggests that SDF-9 and EAK-6 may comprise a PTP that activates downstream signaling through dephosphorylation of tyrosine residues. Although SDF-9 lacks the cysteine residue that is critical for PTP catalytic activity [69], EAK-6 retains this cysteine (Figure 2B) and may possess phosphatase activity. Many receptor-type PTPs harbor tandem catalytic domains, one of which frequently does not have PTP activity [68]. Similarly, SDF-9 and EAK-6 might function as a heteromeric PTP associated with the plasma membrane. The finding that EAK-6 expressed in mammalian cells or bacteria lacks PTP activity on the substrate PNPP does not preclude the possibility that it may have activity on specific phosphotyrosine residues in the context of a full-length protein.

Alternatively, it is possible that EAK-6 has phosphohydrolase activity on nonphosphotyrosine or nonprotein substrates. Among 19 invariant residues in the catalytic domains of 113 vertebrate PTPs [68], 15 are conserved in EAK-6 (Figure 2B). Two of the four invariant residues not conserved in EAK-6, R45 and G220 (PTPN1/PTP1B numbering), are thought to be involved in phosphotyrosine binding [68]. In EAK-6 these are replaced with isoleucine and alanine, respectively. Changes in these residues could reflect changes in EAK-6 substrate specificity.

A third possibility is that EAK-6 does not possess catalytic activity. One of the four invariant residues not conserved in EAK-6, H214, is a glutamine residue in EAK-6 (Figure 2B). This histidine is proposed to lower the pK_a_ of the catalytic cysteine [68]; mutation of H214 to alanine reduces the catalytic activity of PTPN1/PTP1B approximately 80-fold [86]. Two other conserved residues, D181 and Q262, are replaced by glutamate and leucine, respectively, in EAK-6 (Figure 2B); D181E and Q262A mutations reduce PTPN1/PTP1B catalytic activity approximately 600-fold and approximately 80-fold, respectively [86]. Interestingly, the PTPN1/PTP1B H214A mutation reduces K_m_ approximately 5-fold, and the D181E and Q262A mutations reduce K_m_ approximately 10-fold each [86]. Therefore, although substitutions in these conserved residues in EAK-6 may decrease hydrolytic activity, they also may increase binding affinity for substrate. These data are consistent with a model of EAK-6 and SDF-9 functioning as inactive PTP domains that bind to tyrosine phosphoproteins and regulate their interactions with other proteins in a manner similar to STYX family proteins [87]. In this scenario, EAK-6 and SDF-9 could also potentiate DAF-2/InsR signaling by serving as adaptor proteins that increase the local concentration of associated proteins during cascade activation. Resolution of these uncertainties awaits a more detailed analysis of EAK-6 catalytic activity, binding to tyrosine phosphoproteins, and structure.

### Bifurcation of DAF-2/InsR Signaling into Dauer and Longevity Outputs

Temporal and spatial specificity of DAF-2/InsR signaling may underlie differential effects on dauer formation and longevity. Inhibition of DAF-2/InsR signaling during early development enhances dauer formation but has no effect on organismal longevity, and inhibition of DAF-2/InsR signaling in early adulthood is sufficient to extend life span [88]. *eak-4, sdf-9,* and *eak-6* promoter and translational fusions are all expressed continuously from late embryogenesis through early adulthood (unpublished data and [62]); thus, temporal regulation of EAK-4, SDF-9, and EAK-6 expression is not likely to explain the normal life span of *eak-4, sdf-9,* and *eak-6* mutants. The specificity of *eak-4, sdf-9,* and *eak-6* expression in XXX suggests that DAF-2/InsR signaling in the XXX cells may have specific dauer regulatory functions that have no impact on organismal longevity. This model is supported by a recent analysis of tissue-specific functions of DAF-16/FoxO indicating that longevity is primarily regulated by intestinal DAF-16/FoxO, whereas dauer arrest is regulated by neuronal DAF-16/FoxO [89]. Similarly, neuronal DAF-2/InsR regulates life span, whereas intestinal DAF-2/InsR controls metabolism [90]. In light of the observation that DAF-16A::GFP is not expressed in XXX (Figure 6), it will be of great interest to determine whether DAF-16A::GFP localizes to the nucleus in *eak;akt-1* double mutants and, if so, whether there is tissue-specificity of nuclear localization.

### A Model for EAK Protein Function

**Figure 7:**
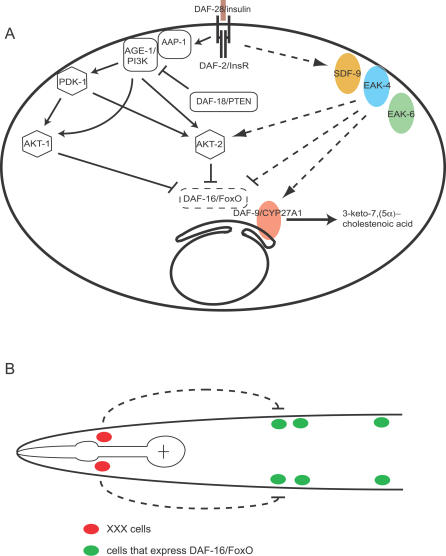
Models of EAK-4, SDF-9, and EAK-6 Function (A) Cell autonomous signaling in XXX. A schematic of one XXX cell is shown. EAK proteins function in parallel with AKT-1 and in the same pathway as AKT-2 to promote nondauer development by potentiating DAF-9/CYP27A1 function either directly or indirectly. DAF-9/CYP27A1 synthesizes 3-keto-7(5α)-cholestenoic acid, a ligand that promotes reproductive development by inhibiting the nuclear hormone receptor DAF-12 [91]. DAF-16/FoxO is denoted with dashed lines, since DAF-16A does not appear to be expressed in XXX. It is not known whether other DAF-16/FoxO isoforms or DAF-2/InsR are expressed in XXX. (B) Nonautonomous signaling from XXX. A schematic of the anterior portion of an animal is shown with the head pointing left. The pharynx is also shown. XXX cells are denoted by red ovals, and DAF-16/FoxO-expressing cells are denoted by green ovals. EAK proteins in the XXX cells generate signals that regulate the synthesis or secretion of a hormone that inhibits DAF-16/FoxO function in other cells.

The data presented in this work are consistent with a model whereby EAK-4, SDF-9, and EAK-6 function in a single complex or pathway at the XXX plasma membrane in parallel with AKT-1 to inhibit dauer formation. Overexpression of the steroid hydroxylase DAF-9/CYP27A1, which is normally expressed in XXX, suppresses dauer arrest in *sdf-9* mutants [62], suggesting that DAF-9 functions downstream of or parallel to SDF-9. In XXX, SDF-9 may function with DAF-9/CYP27A1 to promote the synthesis and/or secretion of dafachronic acids, which are high-affinity ligands for the nuclear hormone receptor DAF-12 [62,91]. Therefore, high EAK protein activity may inhibit dauer formation by potentiating DAF-9/CYP27A1 activity, either directly or indirectly (Figure 7A). EAK proteins may also have nonautonomous inhibitory effects on other cells that express DAF-16/FoxO (Figure 7B). EAK regulation of DAF-16/FoxO probably does not occur through DAF-9/CYP27A1, since *daf-9* is epistatic to *daf-16* for dauer arrest [64,65].

This work identifies two membrane-associated PTP homologs in the endocrine XXX cells that modulate insulin-like signaling in C. elegans. In view of the striking structural and functional similarities in insulin/IGF signaling among metazoa, further studies of insulin/IGF signaling in C. elegans should contribute to our understanding and management of growth factor dysregulation in human disease.

## Materials and Methods

### *eak* mutant isolation, SNP mapping, and sequencing of mutant alleles.

*akt-1(mg306)* animals were mutagenized with ethyl methanesulfonate using standard procedures [92]. Four P_0_ animals were placed on each of 100 6-cm NGM agar plates [93] and removed after overnight egglay at 25 °C. F_2_ dauers were picked to new plates for recovery after visualization under a Nikon SMZ800 dissecting microscope. Recovered dauers were retested for the dauer phenotype at 25 °C. Dauers that bred true were then outcrossed once with N2 (wild-type) animals and subjected to secondary assays. Mutants were tested for suppression by feeding RNAi of *daf-3/SMAD4* and *daf-16/FoxO* as described [56]. Those that were suppressed by *daf-16/FoxO* RNAi but not by *daf-3/SMAD4* RNAi were outcrossed again, and F_2_ dauers were picked for recovery, singled for egglay, and picked to worm lysis buffer containing 50 mM KCl, 10 mM Tris (pH 8.3), 2.5 mM MgCl_2_, 0.45% NP-40, 0.45% Tween 20, 0.01% gelatin, and 60 μg/ml proteinase K. After incubation at −70 °C for at least 10 min, 60 °C for 1 h, and 95 °C for 15 min, 2.5 μl of single worm lysate was PCR-amplified in 100 mM Tris (pH 8.3), 500 mM KCl, 15 mM MgCl_2_, and 0.01% gelatin with Taq polymerase (Roche, Basel, Switzerland) using primers flanking the *akt-1(mg306)* point mutation. *akt-1(mg306)* animals harbor a C-to-T transition at nucleotide 31730 of cosmid C12D8 that creates a TaqI restriction fragment length polymorphism. Mutants for which most or all F_2_ dauers were homozygous for *akt-1(mg306)* were considered true Eak mutants and were analyzed further. All mutants were outcrossed at least four times prior to detailed phenotyping.

SNP mapping was performed essentially as described [67]. Mutants were mated with the Hawaiian C. elegans isolate CB4856, and single F_1_ cross-progeny were allowed to lay eggs overnight at 25 °C. Individual F_2_ dauers were picked for recovery, subjected to egglay, and picked to 100 μl of worm lysis buffer. Lysates were incubated as described above. Then 2.5 μl of single worm lysate was PCR-amplified as described above using genomic primers (Invitrogen, Carlsbad, California, United States) spanning SNPs between CB4856 and the wild-type N2 strain. SNPs were detected by restriction enzyme digestion or sequencing of PCR products.

Genomic fragments corresponding to exons and predicted splice junctions of *eak-4, sdf-9,* and *eak-6* (WormBase Web site, http://www.wormbase.org, release WS120, March 1, 2004) were amplified by PCR and purified using the QIAquick PCR Purification Kit (Qiagen, Valencia, California, United State). Mutations were confirmed by sequencing both strands of DNA.

### Dauer assays.

Two or three gravid animals were picked to individual 6-cm NGM plates, allowed to lay eggs for several hours, and removed. Plates were shifted to the assay temperature, and dauers were scored 48 to 60 h thereafter. All assays of 27 °C dauer arrest were scored in blinded fashion.

### RNAi.

Feeding RNAi was performed as described [94], with minor modifications. Six-well plates containing NGM agar + 5 mM IPTG were spotted with 400 μl of overnight cultures of E. coli HT115 harboring double-stranded RNAi expression plasmid L4440 [95] or L4440 containing *daf-12/NHR-* or *daf-16/FoxO*-specific inserts. Overnight cultures were grown in 2XYT medium containing 50 μg/ml carbenicillin. After overnight incubation of RNAi plates at room temperature (to allow IPTG induction of double-stranded RNA synthesis), two L4 animals were picked to each well, grown at 15 °C, removed after egglay, and shifted to 27 °C. Dauers were scored approximately 60 h thereafter.

### Life span assays.

L4 animals were picked to seeded NGM agar plates containing 0.1 mg/ml 5-fluorodeoxyuridine (ten to 20 animals per plate), incubated at 25 °C, and scored every 1 to 2 d for vitality as described [90]. Animals that did not respond to prodding were scored as dead and removed.

### cDNA isolation.

C. elegans total RNA was isolated as described [96]. cDNA was amplified from total RNA using the SuperScript III RT-PCR Kit (Invitrogen). The 5' and 3' cDNA ends were identified using a 5'/3' RACE kit (Roche). PCR products were cloned into pCR4-TOPO (Invitrogen), and Qiaprep miniprep plasmid DNA (Qiagen) was sequenced using primers flanking the cloned insert.

RT-PCR of wild-type C. elegans total RNA revealed the presence of two alternatively spliced *eak-6* mRNAs differing by the absence or presence of a single exon. The presence of the exon in the long cDNA isoform, *eak-6L,* results in a 17-amino-acid in-frame insertion C-terminal to the PTP domain of EAK-6 (Figure 2A). cDNA analysis also revealed a GeneFinder misprediction in WormBase (WormBase web site, http://www.wormbase.org, release WS120, March 1, 2004) of the exon/intron boundary between exons 3 and 4 (unpublished data). The 5' and 3' RACE identified a 5' cDNA end 13 nucleotides upstream of the translation initiation codon and a 3' cDNA end 303 nucleotides downstream of the translation termination codon but did not identify a *trans*-spliced SL1 leader (unpublished data).

### GFP and RFP reporter constructs.

For promoter fusion constructs, promoter fragments were generated by amplifying 5' upstream sequences between the putative translational start codon and the nearest boundary of the gene immediately upstream. The following promoter fragments were amplified: *eak-4p*: nucleotides 13809 to 14696 of cosmid F53B2 [numbering corresponds to cosmid sequences obtained from the National Center for Biotechnology Information Web site (http://www.ncbi.nlm.nih.gov/)]; *sdf-9p*: nucleotides 10417 to 16170 of YAC Y44A6D; *eak-6p*: nucleotides 4381 to 5426 of cosmid F10G8. PCR primers were tailed with BglII linkers. Promoter fragments were purified using the Qiaquick PCR Purification Kit (Qiagen), digested with BglII, and repurified. *eak-4* and *eak-6* promoter fragments were subcloned into BamHI-digested GFP reporter plasmid pPD95.67 (a gift from Andrew Fire) to generate *eak-4p*::GFP and *eak-6p*::GFP, respectively; the *sdf-9* promoter fragment was subcloned into BamHI-digested RFP [73] reporter plasmid pPD95.75_mRFP3 (a gift from Ho Yi Mak) to generate *sdf-9p*::RFP. Fragment orientation was confirmed by restriction digestion. Plasmids were purified using Qiagen columns.

Protein fusion constructs were made using overlap extension PCR [97,98]. PCR fragments encompassing the putative promoter and open reading frame up to but not including the translation termination codon were fused to a PCR fragment containing GFP and the *unc-54* 3' untranslated region (amplified from the GFP expression vector pPD95.75, a gift from Andrew Fire). The following gene-specific fragments were amplified for protein fusions: EAK-4: nucleotides 12780 to 14696 of cosmid F53B2; SDF-9: nucleotides 10417 to 18862 of YAC Y44A6D; EAK-6: nucleotides 4381 to 7152 of cosmid F10G8. To construct the EAK-4::GFP G2A N-myristoylation mutant, changes were made in the wild-type EAK-4::GFP primers to encode a glycine-to-alanine missense mutation at amino acid 2 of EAK-4. Fusion PCR products were purified using the Qiaquick PCR Purification Kit (Qiagen).

To generate transgenic animals, fusion constructs were coinjected with 1.5–3ng/μl *pha-1*-rescuing plasmid pBX into *pha-1(e2123)* mutant animals, and transgenic animals were selected and maintained by growth at 25 °C [99]. Promoter and protein fusion constructs were injected at approximately 50 ng/μl. In colocalization experiments, 50 ng/μl concentration of either *eak-4p*::GFP or *eak-6p*::GFP was coinjected with 50 ng/μl *sdf-9p*::RFP and 1.5 ng/μl pBX, transgenic lines were established, and colocalization of GFP and RFP was assessed by fluorescence microscopy using a Zeiss Axioplan 2 microscope. In subcellular localization experiments, EAK-4::GFP, SDF-9::GFP, and EAK-6::GFP transgenic lines were established, a strain harboring an integrated *sdf-9p*::RFP array was mated with each GFP strain, and F_1_ animals expressing both GFP and RFP were analyzed by fluorescence microscopy. To generate *daf-2(e1370)* animals harboring the SDF-9::GFP array, *daf-2(e1370)* males were mated with *pha-1(e2123)* animals carrying the SDF-9::GFP *pha-1(+)* array, GFP(+) F_1_ cross-progeny were isolated, and GFP(+) F_2_ dauers were picked after egglay and incubation at 25 °C. Colocalization of DAF-16A::GFP and *sdf-9p*::RFP was analyzed using a Zeiss LSM 510 confocal microscope mounted on a Zeiss Axiovert 100M inverted microscope.

### Rescue experiments.

Cosmid rescue experiments were performed by microinjection of cosmids or cosmid pools into *eak-4(mg326);akt-1(mg306)* double mutant animals and assaying transgenic animals for rescue of dauer formation at 25 °C. SUR-5::GFP was used as a coinjection marker [100]. Cosmids were prepared using the Qiaprep Miniprep Kit (Qiagen) and injected at concentrations of approximately 1 to 10 μg/ml. Single gene rescue experiments were performed with PCR fragments amplified from genomic DNA that contained predicted promoter, open reading frame, and 3' UTR sequences for F53B2.3 *(eak-4)* and F10G8.4 *(eak-6)*. Approximately 10 ng/μl of purified PCR product was coinjected with 100 ng/μl SUR-5::GFP into *eak-4(mg326);akt-1(mg306)* or *eak-6(mg329);akt-1(mg306)* mutant animals, and transgenic animals were assayed for rescue of dauer arrest at 25 °C.

A construct containing the *akt-1* gene under the control of the *eak-4* promoter was made using overlap extension PCR [97,98]. An *eak-4* promoter fragment (nucleotides 13809 to 14696 of cosmid F53B2) was fused to the *akt-1* open reading frame and 3' untranslated region (nucleotides 30791 to 34802 of cosmid C12D8) to create *eak-4p*::AKT-1. Approximately 10 ng/μl *eak-4p*::AKT-1 was coinjected with 100 ng/μl SUR-5::GFP into *eak-4(mg326);akt-1(mg306)* mutant animals, and transgenic (GFP[+]) animals from three independent lines were assayed for rescue of dauer formation at 25 °C. Rescue phenotypes were compared with those of nontransgenic (GFP[−]) siblings.

## Supporting Information

Figure S1 Pharyngeal Morphology and Alae in *akt-1(mg306), eak,* and *eak;akt-1* Dauers *daf-2(e1370)*, *akt-1(mg306)*, *eak-6(mg329)*, and *eak-4(mg348);akt-1(mg306)* animals were grown at 25 °C *(daf-2* and *eak-4;akt-1)* or 27 °C *(akt-1* and *eak-6),* and dauers were examined under Nomarski optics for pharyngeal morphology and the presence of alae. The isthmus and terminal bulb of the pharynx are denoted by arrows, and the alae are denoted by arrowheads. All dauers examined had alae; however, constriction of the isthmus and terminal bulb seen in *daf-2* dauers (top panel) is not observed in *eak-6* or *eak-4;akt-1* dauers (third and bottom panels, respectively). *akt-1* dauers exhibited an intermediate degree of pharyngeal constriction (second panel). *eak-4, sdf-9, akt-1 sdf-9,* and *eak-6;akt-1* dauers had alae and exhibited pharyngeal morphology comparable to *eak-6* and *eak-4;akt-1* dauers.
(2.9 MB PDF)

Figure S2 *eak* Interactions with Mutants in the DAF-2/InsR Signaling Pathway (A) *eak* mutants enhance the dauer arrest phenotype of *age-1/PI3K* and *pdk-1* loss-of-function mutants.
(B) *eak* mutants do not enhance the dauer arrest phenotype of an *akt-2* loss-of-function mutant.
All error bars indicate standard deviation. All experiments were performed twice. Refer to Table S1 for numbers of animals scored.
(826 KB PDF)

Figure S3 Epistasis Analysis of *eak* Mutants with Dauer-Defective *daf-2/InsR* Pathway Mutants (A) *eak;akt-1* 25 °C dauer arrest phenotypes are suppressed by a mutation in *daf-16/FoxO*.
(B) *eak* 27 °C dauer phenotypes are suppressed by a weak loss-of-function mutation in *daf-18/PTEN*.
(C) *eak* 27 °C dauer phenotypes are suppressed by a gain-of-function mutation in *akt-1*. All error bars indicate standard deviation.
All experiments were performed twice. Refer to Table S1 for numbers of animals scored.
(781 KB PDF)

Figure S4 *akt-1, eak,* and *eak;akt-1* Mutants Have Normal Life Spans at 25 °C This experiment was performed twice. Refer to Table S1 for numbers of animals scored.
(553 KB PDF)

Figure S5 *eak-4(mg348)* Is a Null Allele *eak-4(mg348)* males were mated with BC1216 (*sDf21/nT1; +/nT1*) hermaphrodites, and F_1_ cross-progeny were scored for dauer arrest at 27 °C. *sDf21* is a deficiency on chromosome IV that deletes *eak-4*.
(A) F_1_ cross-progeny from *eak-4(mg348)* X BC1216 do not show enhanced dauer arrest at 27 °C compared to *eak-4(mg348)*.
(B) Comparison of observed to expected embryonic lethality among progeny confirms the identity of the BC1216 strain.
(630 KB PDF)

Figure S6 Plasma Membrane Localization of SDF-9::GFP Does Not Require Wild-type DAF-2/InsR Function *daf-2* wild-type and *daf-2(e1370)* L1 animals harboring an SDF-9::GFP translational fusion construct were grown at 25 °C and analyzed using fluorescence microscopy.
(558 KB PDF)

Table S1 Numbers of Animals Assayed (15 KB XLS)

## Abbreviations

GFP: green fluorescent protein
IGF: insulin-like growth factor
PDK: phosphoinositide-dependent kinase
PI: phosphatidylinositol
PKB: protein kinase B
PNPP: *p-nitro*-phenylphosphate
PTP: protein tyrosine phosphatase
SGK: serum- and glucocorticoid-inducible kinase
SNP: single nucleotide polymorphism
TGF: transforming growth factor
