# Supplementary material for: Two Membrane-Associated Tyrosine Phosphatase Homologs Potentiate C. elegans AKT-1/PKB Signaling
Source: PLoS Genet. 2006 Jul 7;2(7):e99. doi: 10.1371/journal.pgen.0020099 (PMC1487177; doi:10.1371/journal.pgen.0020099)
Supplement: Supplementary file 1 [file pgen.0020099.sg001.pdf]

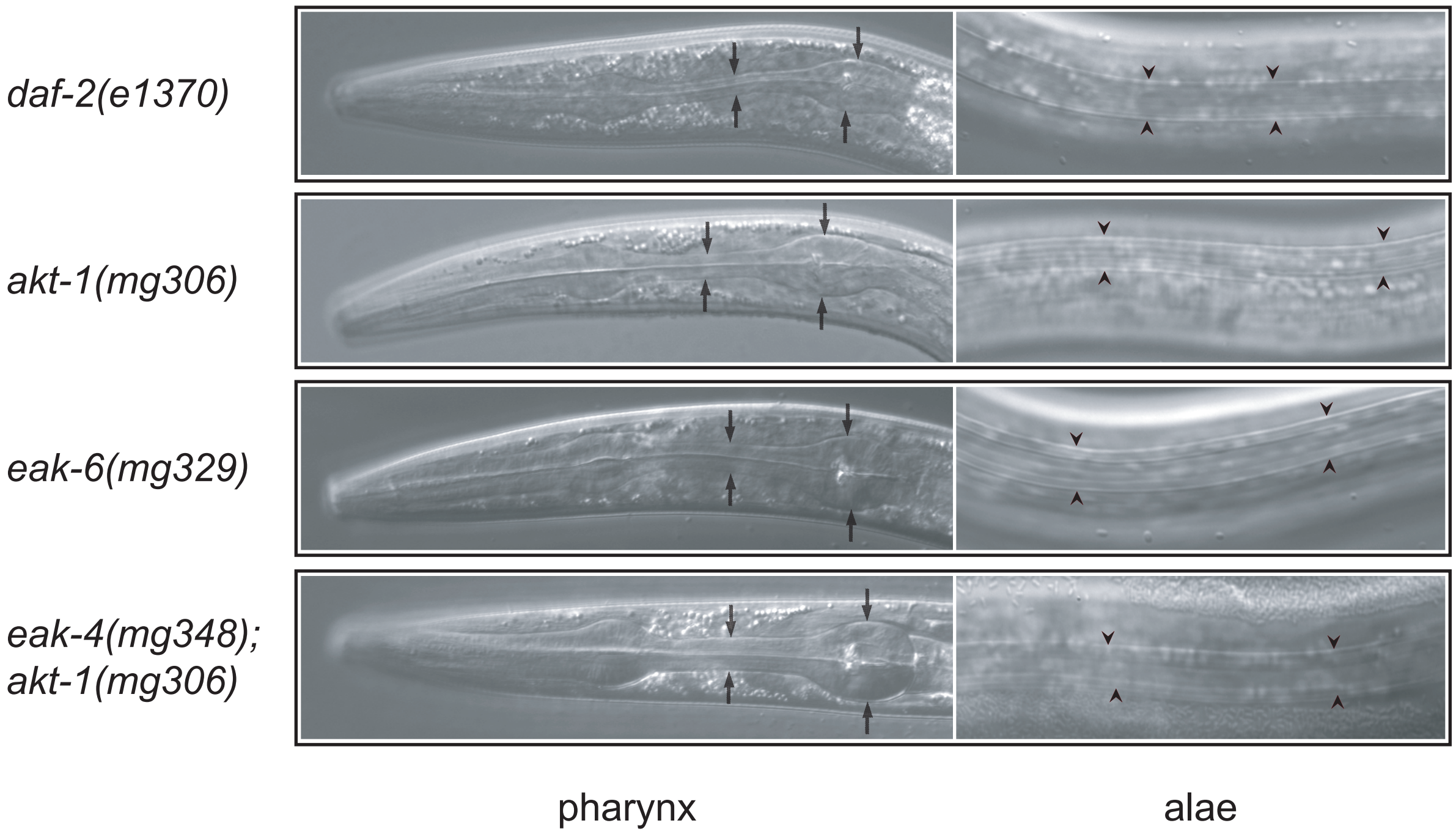
daf-2(e1370)
akt-1(mg306)
eak-6(mg329)
eak-4(mg348); akt-1(mg306)
pharynx
alae
