# Supplementary material for: Two Membrane-Associated Tyrosine Phosphatase Homologs Potentiate C. elegans AKT-1/PKB Signaling
Source: PLoS Genet. 2006 Jul 7;2(7):e99. doi: 10.1371/journal.pgen.0020099 (PMC1487177; doi:10.1371/journal.pgen.0020099)
Supplement: Supplementary file 2 [file pgen.0020099.sg002.pdf]

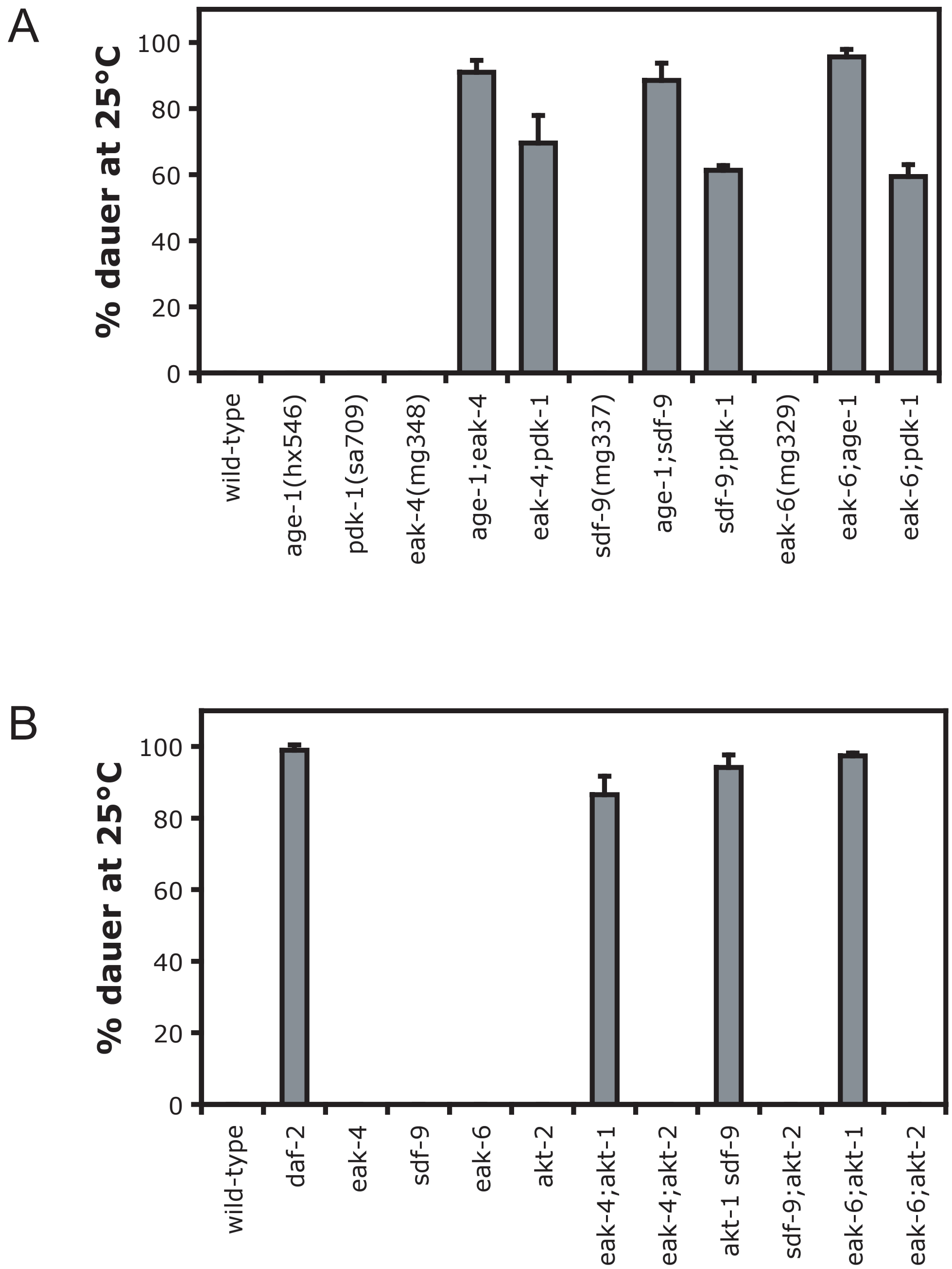
A
% dauer at 25°C
100
80
60
40
20
0
wild-type
age-1(hx546)
pdk-1(sa709)
eak-4(mg348)
age-1;eak-4
eak-4;pdk-1
sdf-9(mg337)
age-1;sdf-9
sdf-9;pdk-1
eak-6(mg329)
eak-6;age-1
eak-6;pdk-1
B
% dauer at 25°C
100
80
60
40
20
0
wild-type
daf-2
eak-4
sdf-9
eak-6
akt-2
eak-4;akt-1
eak-4;akt-2
akt-1 sdf-9
sdf-9;akt-2
eak-6;akt-1
eak-6;akt-2
