# Supplementary material for: Two Membrane-Associated Tyrosine Phosphatase Homologs Potentiate C. elegans AKT-1/PKB Signaling
Source: PLoS Genet. 2006 Jul 7;2(7):e99. doi: 10.1371/journal.pgen.0020099 (PMC1487177; doi:10.1371/journal.pgen.0020099)
Supplement: Supplementary file 3 [file pgen.0020099.sg003.pdf]

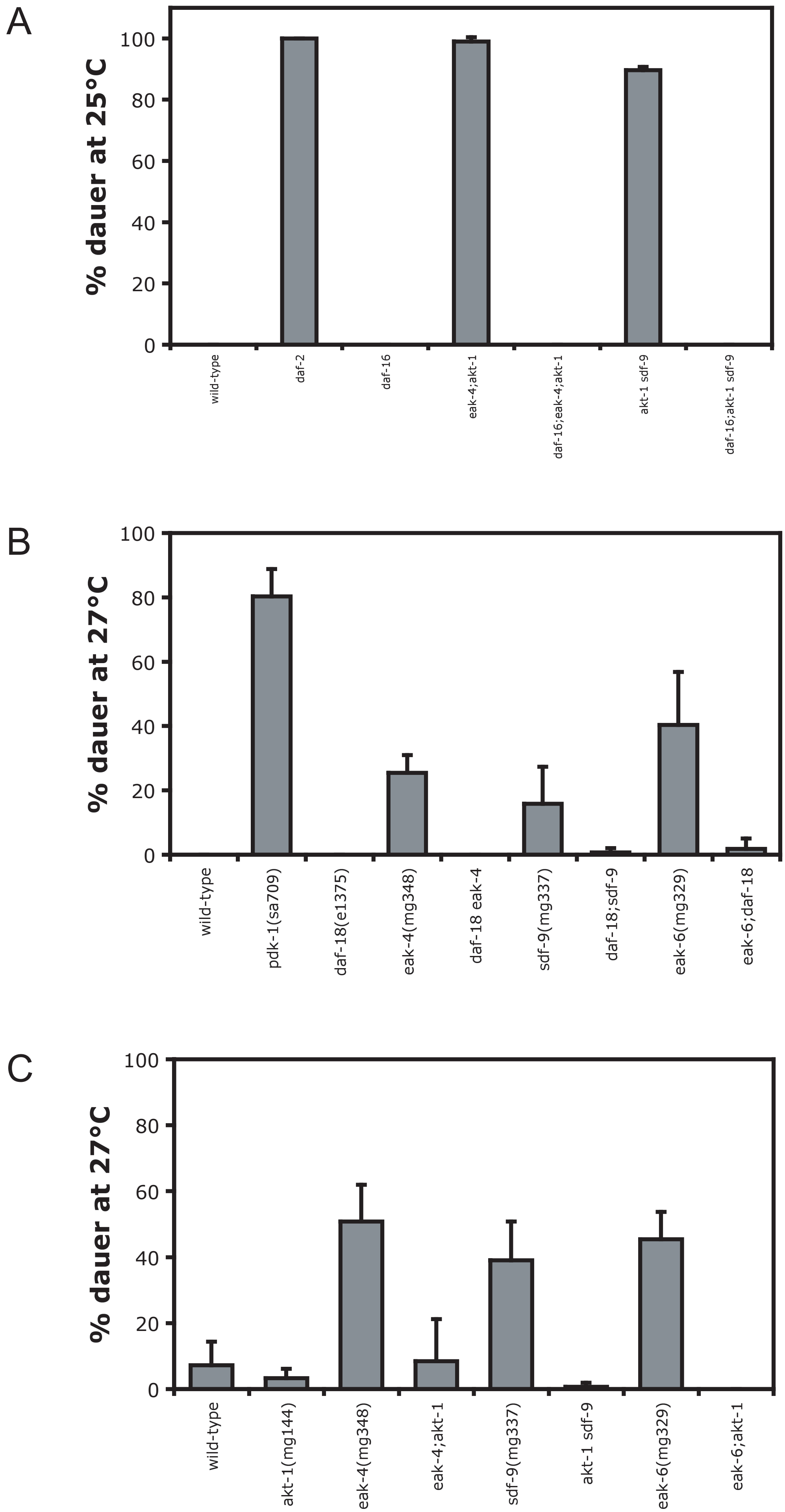
A
% dauer at 25°C
0
20
40
60
80
100
wild-type
daf-2
daf-16
eak-4;akt-1
daf-16;eak-4;akt-1
akt-1 sdf-9
daf-16;akt-1 sdf-9
B
% dauer at 27°C
0
20
40
60
80
100
wild-type
pdk-1(sa709)
daf-18(e1375)
eak-4(mg348)
daf-18 eak-4
sdf-9(mg337)
daf-18;sdf-9
eak-6(mg329)
eak-6;daf-18
C
% dauer at 27°C
0
20
40
60
80
100
wild-type
akt-1(mg144)
eak-4(mg348)
eak-4;akt-1
sdf-9(mg337)
akt-1 sdf-9
eak-6(mg329)
eak-6;akt-1
