# Supplementary material for: Two Membrane-Associated Tyrosine Phosphatase Homologs Potentiate C. elegans AKT-1/PKB Signaling
Source: PLoS Genet. 2006 Jul 7;2(7):e99. doi: 10.1371/journal.pgen.0020099 (PMC1487177; doi:10.1371/journal.pgen.0020099)
Supplement: Supplementary file 4 [file pgen.0020099.sg004.pdf]

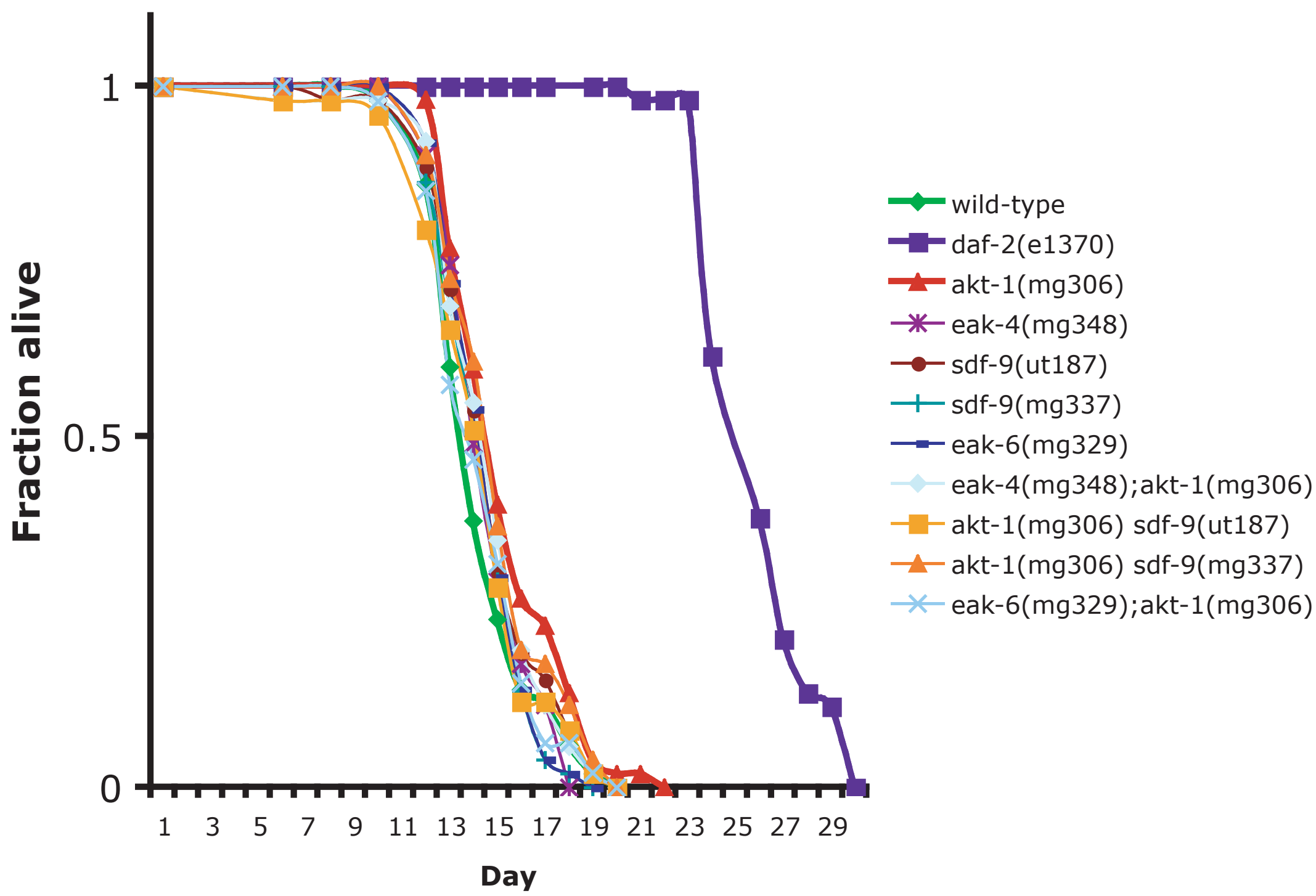

Fraction alive
1
0.5
0
1
3
5
7
9
11
13
15
17
19
21
23
25
27
29
Day
wild-type
daf-2(e1370)
akt-1(mg306)
eak-4(mg348)
sdf-9(ut187)
sdf-9(mg337)
eak-6(mg329)
eak-4(mg348);akt-1(mg306)
akt-1(mg306) sdf-9(ut187)
akt-1(mg306) sdf-9(mg337)
eak-6(mg329);akt-1(mg306)
