# Supplementary material for: Two Membrane-Associated Tyrosine Phosphatase Homologs Potentiate C. elegans AKT-1/PKB Signaling
Source: PLoS Genet. 2006 Jul 7;2(7):e99. doi: 10.1371/journal.pgen.0020099 (PMC1487177; doi:10.1371/journal.pgen.0020099)
Supplement: Supplementary file 5 [file pgen.0020099.sg005.pdf]

A

B

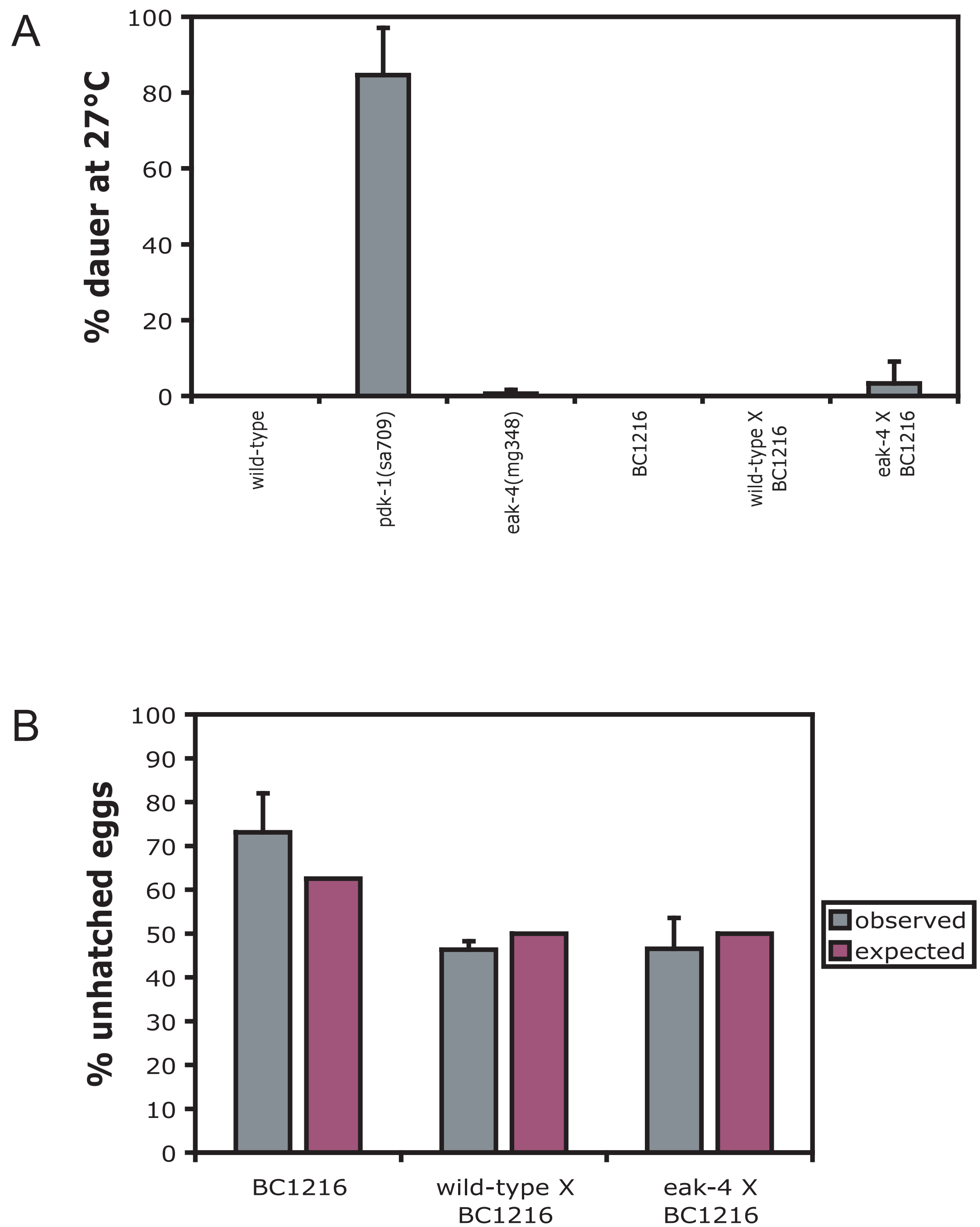
A
% dauer at 27°C
0
20
40
60
80
100
wild-type
pdk-1(sa709)
eak-4(mg348)
BC1216
wild-type X BC1216
eak-4 X BC1216
B
% unhatched eggs
0
10
20
30
40
50
60
70
80
90
100
BC1216
wild-type X BC1216
eak-4 X BC1216
observed
expected
