# Supplementary material for: Two Membrane-Associated Tyrosine Phosphatase Homologs Potentiate C. elegans AKT-1/PKB Signaling
Source: PLoS Genet. 2006 Jul 7;2(7):e99. doi: 10.1371/journal.pgen.0020099 (PMC1487177; doi:10.1371/journal.pgen.0020099)
Supplement: Supplementary file 6 [file pgen.0020099.sg006.pdf]

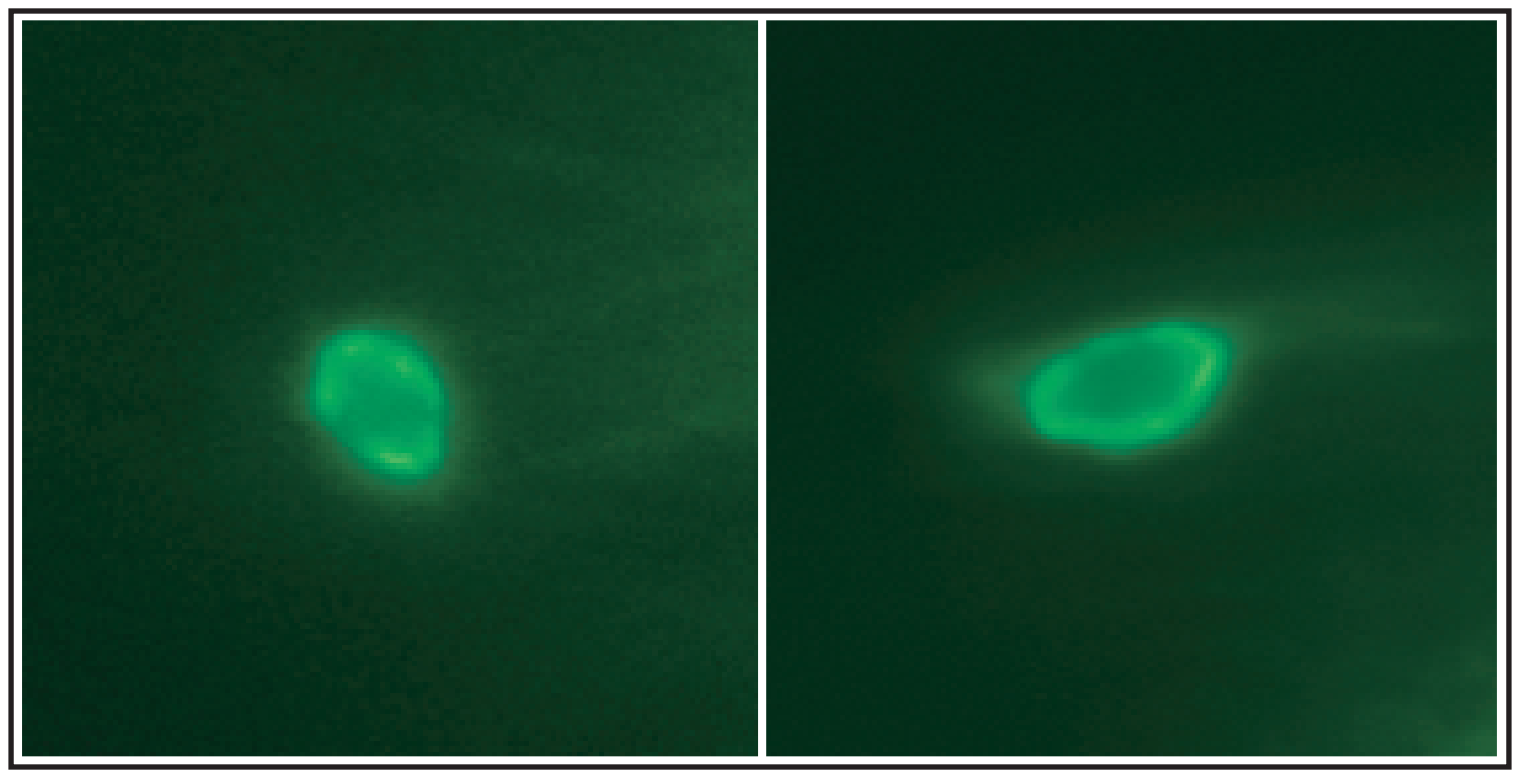

wild-type *e1370*
